# Long-term immunological impairment after early thymectomy in congenital heart disease

**DOI:** 10.1007/s12026-026-09858-z

**Published:** 2026-10-07

**Authors:** José Roberto Mendes Pegler, Débora Linhares Rodrigues, Renata de Almeida Bordim, Patricia Palmeira, Andréia Rangel-Santos, Isabela de Jesus Silva Avelar, Ana Cristina Sayuri Tanaka, Estela Azeka, Nana Miura Ikari, Magda Maria Sales Carneiro-Sampaio, Marcelo Biscegli Jatene, Antonio Carlos Pastorino

**Affiliations:** 1 Departamento de Pediatria, Faculdade de Medicina FMUSP, Universidade de São Paulo, São Paulo, SP Brazil; 2 Instituto da Criança e do Adolescente (ICr), Hospital das Clínicas HCFMUSP, Faculdade de Medicina, Universidade de São Paulo, Av. Dr. Enéas de Carvalho Aguiar, 647, Cerqueira César, São Paulo, SP 05403-000 Brazil; 3 Laboratório de Pediatria Clínica (LIM-36), Faculdade de Medicina, Hospital das Clínicas HCFMUSP, Universidade de São Paulo, São Paulo, SP Brazil; 4 Instituto Do Coração (InCor), Hospital das Clínicas HCFMUSP, Faculdade de Medicina, Universidade de São Paulo, São Paulo, SP Brazil

**Keywords:** Congenital heart disease, Thymectomy, Naïve T cells, Regulatory T cells, TREC

## Abstract

Early thymectomy during congenital heart disease repair may impair thymic output, but long-term effects across age groups remain incompletely defined. We evaluated T-cell receptor excision circles (TRECs) and a comprehensive panel of lymphocyte subsets in 20 adults (mean age, 29.6 years) and 16 children (mean age, 10.7 years) who underwent thymectomy within the first year of life, and in age- and sex-matched controls. TRECs were lower in patients than controls in adults (34 ± 25 vs. 79 ± 45 copies/μL; FDR-adjusted *p* = 0.001) and children (9 [7–15] vs. 95 [65–160] copies/μL; FDR-adjusted *p* < 0.0001). Total lymphocyte and T-cell counts were reduced in both age groups, mainly due to lower naïve CD4⁺ and CD8⁺ T cells, including recent thymic emigrants. Most non-naïve subsets did not differ significantly from controls. Regulatory T-cell (Treg) alterations differed by age: children showed broad reductions, whereas adults showed selective naïve Treg reduction. Early thymectomy is associated with persistent thymic output impairment, including in adults approximately three decades after surgery, and with age-specific Treg alterations.

**Supplementary Information:**

The online version contains supplementary material available at https://doi.org/10.1007/s12026-026-09858-z.

## Introduction

Over the last decades, the number of people living with congenital heart disease (CHD) has progressively increased. Due to advances in treatment, most individuals born with CHD (which affects approximately 1–2% of live births) now survive well into adulthood, and approximately 70% of those surviving to age 18 live beyond 70 years [1]. Critical heart defects will typically require surgical intervention within the first year of life and account for approximately 25% of cases. Advances in pediatric cardiac surgery have substantially improved survival and long-term life expectancy in this population [2].

The thymus is the primary lymphoid organ responsible for the maturation of T lymphocytes. It is highly active during fetal life and early childhood, followed by a gradual decline in naïve T-cell output with aging due to thymic involution [3]. During the first months of life, the thymus is relatively large compared with other mediastinal structures, including the great vessels. Therefore, partial or complete thymectomy has traditionally been performed during surgical correction of complex congenital heart disease to improve exposure of the surgical field [4].

The first studies evaluating the long-term immunological repercussions of early thymectomy were published nearly four decades ago [5], yet these consequences remain incompletely understood. Several studies have consistently shown that the T-cell compartment is diminished in patients who underwent thymectomy during early surgical correction of CHD, mostly due to a reduction in naïve T-cell subsets in younger children [6, 7], older children and adolescents [8, 9], and adults [10, 11]. More detailed analyses of the T-cell compartment, including recent thymic emigrants, mature naïve T cells, distinct memory subsets, and regulatory T cells (Tregs), remain limited in the published literature. T-cell receptor excision circles (TRECs), which are generated during T-cell receptor rearrangement in the thymus, provide an indirect measure of thymic output and have also been shown to be reduced following early thymectomy [12, 13].

However, data regarding the complex long-term immunological consequences of early thymectomy across different age groups, particularly in adults and in Latin American populations, remain scarce.

The aim of this study was to evaluate the cellular immune compartment in children and adults who underwent early thymectomy during surgical repair of critical CHD at a tertiary university hospital in Brazil and to compare them with age-matched controls, years after surgery.

## Materials and methods

### Study design and population

Patients with complex CHD from the Heart Institute (InCor), Hospital das Clínicas, University of São Paulo, Brazil, who underwent thymectomy during cardiac surgical repair within the first year of life were recruited to participate in this cross-sectional study.

Two different age groups were included: adults (≥ 18 years) and children (aged 6–13 years). Individuals without congenital heart disease or a history of thoracic surgery or thymectomy were recruited as controls and frequency-matched to patients by age and sex within each age group.

Because information regarding the extent of thymectomy was frequently unavailable in older medical records, only patients with transposition of the great arteries (TGA) or with double-outlet right ventricle (DORV), who underwent surgery with the Jatene procedure (in which thymectomy is typically required to optimize surgical exposure of the great arteries) were selected.

Every participant was informed about the study through a telephone call and/or text message, invited for an interview and for blood sample collection, and at this time written informed consent was obtained.

Exclusion criteria were any diagnosis or clinical features suggestive of congenital syndromes (such as 22q11.2 deletion syndrome or others), acute infections requiring antibiotics or hospitalization within 30 days prior to blood collection, history of cardiac transplantation, inborn errors of immunity or use of any immunosuppressive treatment.

Participants were recruited in Brazil, and the cohort is therefore described as Brazilian/Latin American based on geographic and clinical recruitment. Genetic ancestry testing was not performed, and self-reported race/color or ethnicity was not systematically collected.

### Laboratory analysis

#### Immunophenotyping of lymphocyte subsets

Flow cytometry analysis of lymphocyte subsets was performed in peripheral blood collected in EDTA tubes. Red blood cells were lysed using BD Pharm Lyse™ buffer (BD Biosciences, San Jose, CA, USA), followed by washing with FACSFlow™ solution (BD Biosciences). Cells were then resuspended in FACSFlow at a concentration of 1 × 10⁶ cells/mL and incubated with fluorochrome-conjugated monoclonal antibodies for 30 min at room temperature in the dark. For intracellular FOXP3 staining, cells were fixed and permeabilized using Cytofix/Cytoperm™ (BD Biosciences), followed by intracellular staining with anti-FOXP3 and anti-CTLA-4 antibodies. After staining, cells were washed and finally resuspended in 300 μL of FACSFlow for acquisition. Details regarding monoclonal antibody specificity, fluorochrome, clone, and volume used in the flow cytometry experiments are provided in Supplementary Table S1.

Data acquisition was performed using a BD LSR II Fortessa™ flow cytometer and BD FACSDiva™ software, and analyses were conducted using FlowJo software (TreeStar, Ashland, OR, USA). Absolute cell counts were calculated by multiplying the percentage of each population obtained by flow cytometry by the absolute lymphocyte count determined using an automated hematology analyzer (Sysmex XP-300, Sysmex Corporation, Kobe, Japan).

#### T-cell receptor excision circle (TREC) quantification

Genomic DNA was extracted from peripheral blood using the QIAamp DNA Blood Kit (Qiagen, Valencia, CA, USA) according to the manufacturer’s instructions. TREC copy numbers were quantified by real-time quantitative polymerase chain reaction (qPCR) using the StepOnePlus™ Real-Time PCR System (Applied Biosystems, Foster City, CA, USA) and TaqMan® Gene Expression Master Mix (Applied Biosystems, USA). PCR reactions were performed in a final volume of 25 μL using TREC- or ACTB-specific oligonucleotides and TREC (6FAM-TAMRA) or ACTB (6VIC-TAMRA) probes, prepared at concentrations of 20 μM and 15 μM, respectively (Integrated DNA Technologies, USA). All reactions were performed in triplicate. ACTB amplification was used as an endogenous control for DNA quality assessment and normalization.

The thermal cycling conditions consisted of an initial incubation at 50 °C for 2 min and 95 °C for 10 min, followed by 40 cycles of 95 °C for 30 s and 60 °C for 1 min. A calibration curve was included in each qPCR run for absolute quantification of TREC copies and was generated using serial 10-fold dilutions (10^2^–10⁶ copies/μL) of plasmids containing the TREC sequence. TREC results were expressed as copies/μL of peripheral blood.

### Statistics

Demographic and clinical characteristics were summarized using descriptive statistics. The distribution of quantitative variables was assessed using the Shapiro–Wilk test. Variables with a normal distribution were expressed as mean ± standard deviation (SD), whereas non-normally distributed variables were expressed as median and interquartile range (IQR). Qualitative variables were described using absolute frequencies.

Comparisons between patients and controls within each age group were performed using Student’s t-test for normally distributed continuous variables and the Mann–Whitney U test for non-normally distributed continuous variables. To account for multiple testing, *p*-values obtained from comparisons within each age group were adjusted using the Benjamini–Hochberg false discovery rate (FDR) procedure. Statistical significance was defined as an FDR-adjusted *p*-value <0.05. Both raw *p*-values and FDR-adjusted *p*-values are provided in Supplementary Table S2. Exploratory correlations between age at thymectomy and immunological parameters were assessed using Spearman’s rank correlation coefficient in thymectomized patients, separately within each age group. *P*-values from these exploratory correlation analyses were also adjusted using the Benjamini–Hochberg FDR procedure. All analyses were conducted using MedCalc software (version 20.218; MedCalc Software Ltd, Ostend, Belgium).

## Results

### Patient demographics

Demographic characteristics of the study population are summarized in Table 1. As expected from the frequency-matching procedure, patients and controls had similar age and sex distributions within each age group. There was no statistically significant difference in age at thymectomy between adult and pediatric patients (*p* = 0.16). Exploratory Spearman correlation analyses did not identify consistent associations between age at thymectomy and immunological parameters after correction for multiple comparisons.

**Table 1:** Demographic and clinical characteristics of adult and pediatric patients and controls

	Adult Patients (AP)	Adult Controls (AC)	Pediatric Patients (PP)	Pediatric Controls (PC)
n	20	20	16	16
Sex (Male/Female)	13/7	13/7	15/1	15/1
Age (years)	29.6 ± 3.8	29.9 ± 3.6	10.7 ± 2.2	10.2 ± 2.3
Age at thymectomy (days)	12 (7–110)	—	11 (4–18)	—
Cardiac diagnosis				
TGA	16	—	16	—
DORV	4	—	0	—

Data are presented as mean ± SD or median (IQR). *TGA* Transposition of the Great Arteries; *DORV* Double-outlet Right Ventricle

### TREC quantification

The mean TREC level was significantly lower in adult patients (AP) than in adult controls (AC) (34 ± 25 vs. 79 ± 45 copies/μL, respectively; FDR-adjusted *p* = 0.001).

The median TREC level was significantly lower in pediatric patients (PP) than in pediatric controls (PC) (9 copies/μL [IQR 7–15] vs. 95 copies/μL [IQR 65–160], respectively; FDR-adjusted *p* < 0.0001).

**Fig. 1:**
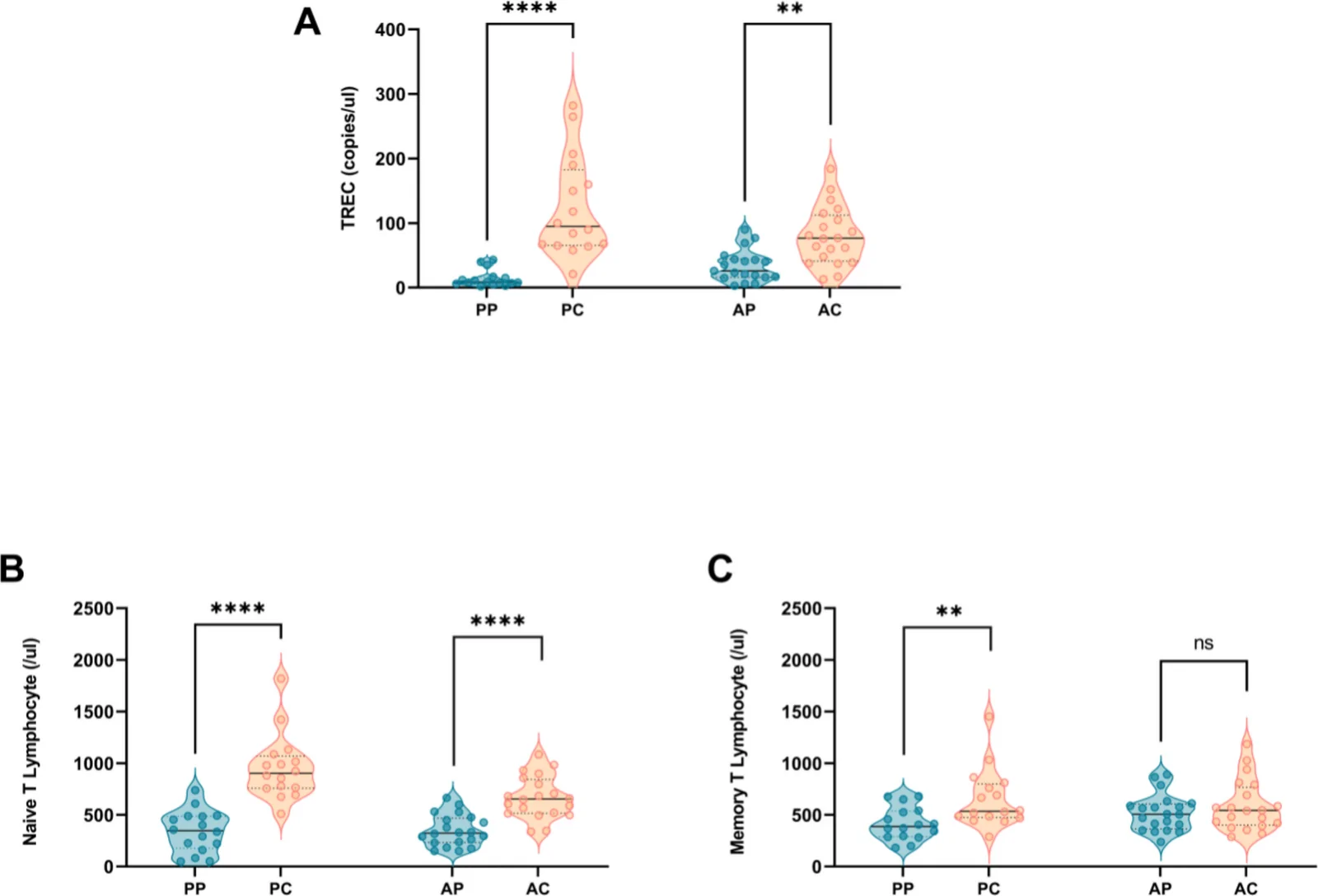
TREC levels and selected T-cell subsets in thymectomized patients and healthy controls. Violin plots with individual data points showing (**A**) T-cell receptor excision circles (TRECs), (**B**) naïve T lymphocytes, and (**C**) memory T lymphocytes in pediatric thymectomized patients (PP), pediatric controls (PC), adult thymectomized patients (AP), and adult controls (AC). TREC values are expressed as copies/µL, and lymphocyte subsets are expressed as cells/µL. Horizontal lines indicate summary measures within each group. Statistical comparisons were performed between thymectomized patients and their respective age-matched controls using FDR-adjusted *p*-values. **p < 0.01; ****p < 0.0001; ns, not significant

Individual-value plots illustrating TREC levels in pediatric and adult thymectomized patients and controls are shown in Fig. 1A.

### Lymphocyte analysis

#### Total lymphocytes and T-cell subsets

The results of the flow cytometric analysis of total lymphocytes, T cells (CD3⁺), and their subsets are summarized in Table 2.

**Table 2:** Analysis of total lymphocytes, T cells, and T-cell subsets

Lymphocyte subset (phenotypic markers)	AP (*n* = 20)	AC (*n* = 20)	FDR-adjusted p	PP (*n* = 16)	PC (*n* = 16)	FDR-adjusted p
Total lymphocytes	1790 ± 453	2275 ± 504	** < 0.01**	1806 ± 643	2682 ± 627	**0.001**
Total T cells (CD3⁺)	1019 ± 333	1523 ± 409	** < 0.001**	870 (660–1055)	1558 (1350–1709)	** < 0.0001**
Naïve T cells (CD3⁺CD45RA⁺)	350 ± 152	670 ± 204	** < 0.0001**	340 ± 202	955 ± 315	** < 0.0001**
RTE T cells (CD3⁺CD45RA⁺CD27⁺CCR7⁺CD62L⁺CD31⁺)	72 ± 73	251 ± 105	** < 0.0001**	77 (23–142)	339 (262–404)	** < 0.001**
Mature naïve T cells (CD3⁺CD45RA⁺CD27⁺CCR7⁺CD62L⁺CD31⁻)	33 (10–82)	105 (59–139)	** < 0.01**	60 ± 51	165 ± 80	** < 0.001**
Memory T cells (CD3⁺CD45RO⁺)	522 ± 178	589 ± 247	*0.42*	388 (286–526)	536 (470–761)	** < 0.01**

All values are expressed as cells/μL. Data are presented as mean ± SD or median (IQR), according to distribution. *P*-values shown are Benjamini–Hochberg false discovery rate (FDR)–adjusted *p*-values. Statistical significance was defined as FDR-adjusted *p* < 0.05. *AP* adult patients; *AC* adult controls; *PP* pediatric patients; *PC* pediatric controls; *RTE* recent thymic emigrant

Total lymphocyte counts were significantly lower in both adult and pediatric patients than in their respective control groups. The reduction in total lymphocyte counts was largely accounted for by lower total T-cell counts in both age groups.

In addition to reduced total lymphocyte and T-cell counts, all naïve T-cell subsets were significantly reduced in patients from both age groups. In contrast, total memory T-cell counts differed significantly from controls only in pediatric patients.

Individual-value plots of selected T-cell subsets, including naïve and memory T cells, are shown in Fig. 1B–C, illustrating the distribution of values and the magnitude of differences between thymectomized patients and controls.

#### Total and naïve CD4⁺ and CD8⁺ subsets

The results of the flow cytometric analysis of total CD4⁺ and CD8⁺ T cells and their naïve subsets are summarized in Table 3.

**Table 3:** Analysis of total CD4⁺ and CD8⁺ T cells and their naïve subsets

Lymphocyte subset (phenotypic markers)	AP (*n* = 20)	AC (*n* = 20)	FDR-adjusted p	PP (*n* = 16)	PC (*n* = 16)	FDR-adjusted p
Total CD4⁺ T cells (CD3⁺CD4⁺)	577 ± 181	804 ± 269	** < 0.01**	481 (364–524)	809 (693–909)	** < 0.0001**
Naïve CD4⁺ T cells (CD3⁺CD4⁺CD45RA⁺)	144 (108–176)	293 (229–347)	** < 0.01**	80 (53–135)	436 (367–518)	** < 0.0001**
CD4⁺ naïve RTE T cells (CD3⁺CD4⁺CD45RA⁺CD27⁺CCR7⁺CD62L⁺CD31⁺)	30 (8–56)	100 (76–161)	** < 0.001**	40 ± 40	169 ± 94	** < 0.0001**
CD4⁺ mature naïve T cells (CD3⁺CD4⁺CD45RA⁺CD27⁺CCR7⁺CD62L⁺CD31⁻)	37 (9–59)	84 (37–107)	** < 0.01**	21 (11–43)	95 (80–103)	** < 0.001**
Total CD8⁺ T cells (CD3⁺CD8⁺)	244 (185–330)	453 (403–530)	** < 0.001**	288 (170–351)	460 (357–512)	** < 0.001**
Naïve CD8⁺ T cells (CD3⁺CD8⁺CD45RA⁺)	103 (72–137)	209 (161–294)	** < 0.001**	114 (38–162)	236 (180–300)	** < 0.0001**

All values are expressed as cells/μL. Data are presented as mean ± SD or median (IQR), according to distribution. *P*-values shown are Benjamini–Hochberg false discovery rate (FDR)–adjusted *p*-values. Statistical significance was defined as FDR-adjusted *p* < 0.05. *AP* adult patients; *AC* adult controls; *PP* pediatric patients; *PC* pediatric controls; *RTE* recent thymic emigrant

Total CD4⁺ and CD8⁺ T cells, as well as all naïve subsets, were significantly reduced in patients from both age groups.

#### Non-naïve CD4⁺ and CD8⁺ subsets

The results of the flow cytometric analysis of non-naïve CD4⁺ and CD8⁺ T-cell subsets are summarized in Table 4.

**Table 4:** Analysis of non-naïve CD4⁺ and CD8⁺ T-cell subsets

Lymphocyte subset (phenotypic markers)	AP (*n* = 20)	AC (*n* = 20)	FDR-adjusted p	PP (*n* = 16)	PC (*n* = 16)	FDR-adjusted p
Activated CD4⁺ T cells (CD3⁺CD4⁺HLA-DR⁺)	45 (33–66)	51 (36–75)	*0.70*	25 (11–74)	34 (21–42)	*0.74*
CD4⁺ memory T cells (CD3⁺CD4⁺CD45RO⁺)	318 ± 111	372 ± 138	*0.28*	292 ± 90	369 ± 130	*0.08*
CD4⁺ TCM (CD3⁺CD4⁺CD45RO⁺CD27⁺CCR7⁺CD62L⁺)	74 ± 38	103 ± 62	*0.15*	132 ± 55	123 ± 62	*0.71*
CD4⁺ TTM (CD3⁺CD4⁺CD45RO⁺CD27⁺CCR7⁻CD62L⁻)	21 (15–30)	32 (19–48)	*0.28*	12 (10–18)	22 (13–26)	**0.02**
CD4⁺ TEM(CD3⁺CD4⁺CD45RO⁺CD27⁻CCR7⁻CD62L⁻)	47 (30–59)	52 (37–67)	*0.54*	26 ± 15	48 ± 29	**0.02**
CD4⁺ TEMRA cells (CD3⁺CD4⁺CD45RA⁺CCR7⁻)	41 (21–55)	56 (31–94)	*0.20*	19 (9–28)	71 (46–114)	**0.0001**
Senescence CD4⁺ T cells (CD3⁺CD4⁺CD45RO⁻CD27⁻CD28⁻CD57⁺)	4 (2.8–12.5)	4.1 (1.7–7)	*0.54*	2.7 (0.7–8.7)	3.5 (1.1–8.2)	*0.71*
Exhaustion-associated CD4⁺ memory T cells (CD3⁺CD4⁺CD45RO⁺CD27⁻PD-1⁺)	95 (64–139)	96 (55–159)	*0.95*	45 (28–50)	37 (15–44)	*0.36*
Activated CD8⁺ T cells (CD3⁺CD8⁺HLA-DR⁺)	27 (15–58)	37 (27–49)	*0.28*	16 (6–28)	20 (14–33)	*0.25*
CD8⁺ memory T cells (CD3⁺CD8⁺CD45RO⁺)	107 (80–157)	179 (111–210)	**0.01**	117 (89–143)	164 (125–199)	**0.03**
CD8⁺ TCM (CD3⁺CD8⁺CD45RO⁺CD27⁺CCR7⁺CD62L⁺)	12 (9–26)	17 (9–34)	*0.54*	18 (12–32)	31 (24–40)	*0.08*
CD8⁺ TTM (CD3⁺CD8⁺CD45RO⁺CD27⁺CCR7⁻CD62L⁻)	15 (8–22)	21 (17–37)	**0.02**	12 (10–20)	14 (7–20)	*0.98*
CD8⁺ TEM (CD3⁺CD8⁺CD45RO⁺CD27⁻CCR7⁻CD62L⁻)	14 (3–19)	22 (11–35)	*0.09*	7.7 (4.7–16.1)	16 (8–26)	**0.04**
CD8⁺ TEMRA cells (CD3⁺CD8⁺CD45RA⁺CCR7⁻)	69 ± 44	116 ± 70	**0.03**	52 (14–89)	101 (63–154)	** < 0.01**
Senescence CD8⁺ T cells (CD3⁺CD8⁺CD45RO⁻CD27⁻CD28⁻CD57⁺)	27 (13–77)	30 (10–71)	*0.95*	16 (5–28)	29 (10–43)	*0.16*
Exhaustion-associated CD8⁺ memory T cells (CD3⁺CD8⁺CD45RO⁺CD27⁻PD-1⁺)	43 (25–76)	32 (19–65)	*0.42*	14 (5–21)	7.8 (5.6–18)	*0.86*

All values are expressed as cells/μL. Data are presented as mean ± SD or median (IQR), according to distribution. *P*-values shown are Benjamini–Hochberg false discovery rate (FDR)–adjusted *p*-values. Statistical significance was defined as FDR-adjusted *p* < 0.05. *AP* adult patients; *AC* adult controls; *PP* pediatric patients; *PC* pediatric controls; *TCM* central memory T cells; *TTM* transitional memory T cells; *TEM* effector memory T cells; *TEMRA* terminally differentiated effector memory T cells re-expressing CD45RA

Most non-naïve CD4⁺ and CD8⁺ T-cell subsets did not differ significantly between adult patients and controls, with significant differences observed in only 3 of the 16 subsets analyzed, all within the CD8⁺ non-naïve compartment. A similar pattern was observed in the pediatric group, in which 10 of the 16 non-naïve subsets did not differ significantly between patients and controls.

#### Regulatory T cells

The results of the flow cytometric analysis of regulatory T cells (Tregs) and their subsets are summarized in Table 5.

**Table 5:** Analysis of regulatory T cells

Lymphocyte subset (phenotypic markers)	AP (*n* = 20)	AC (*n* = 20)	FDR-adjusted p	PP (*n* = 16)	PC (*n* = 16)	FDR-adjusted p
Total Tregs(CD3⁺CD4⁺CD127⁻CD25⁺FoxP3⁺)	18 ± 8	23 ± 12	*0.20*	2.2 (1.3–3.2)	25 (14–37)	** < 0.0001**
Naïve Tregs (CD3⁺CD4⁺CD127⁻CD25⁺FoxP3⁺CD45RA⁺)	3.3 (2.3–4.7)	5.9 (4–9.8)	**0.01**	0.04 (0.02–0.12)	10.2 (6.8–12.6)	** < 0.0001**
CTLA-4⁺ Tregs (CD3⁺CD4⁺CD127⁻CD25⁺FoxP3⁺CTLA-4⁺)	1.6 (1.2–2.8)	1.9 (1–3.7)	*0.62*	0.5 (0.2–1)	5.2 (3.8–9.7)	** < 0.0001**
CD39⁺ Tregs (CD3⁺CD4⁺CD127⁻CD25⁺FoxP3⁺CD39⁺)	4.9 (2.5–7.9)	3.1 (2–6.2)	*0.42*	0.8 (0.5–1.2)	5.3 (3.9–6)	** < 0.0001**

All values are expressed as cells/μL. Data are presented as mean ± SD or median (IQR), according to distribution. *P*-values shown are Benjamini–Hochberg false discovery rate (FDR)–adjusted *p*-values. Statistical significance was defined as FDR-adjusted *p* < 0.05. *AP* adult patients; *AC* adult controls; *PP* pediatric patients; *PC* pediatric controls; *Tregs* regulatory T cells

In adult patients, only the naïve Treg subset was significantly reduced, whereas total Tregs and Treg subsets expressing suppression-associated markers (CTLA-4⁺ and CD39⁺) did not differ significantly from controls. In contrast, pediatric patients exhibited significantly lower counts of total Tregs and all Treg subsets than controls, with the most pronounced reduction observed in the naïve Treg subset.

**Fig. 2:**
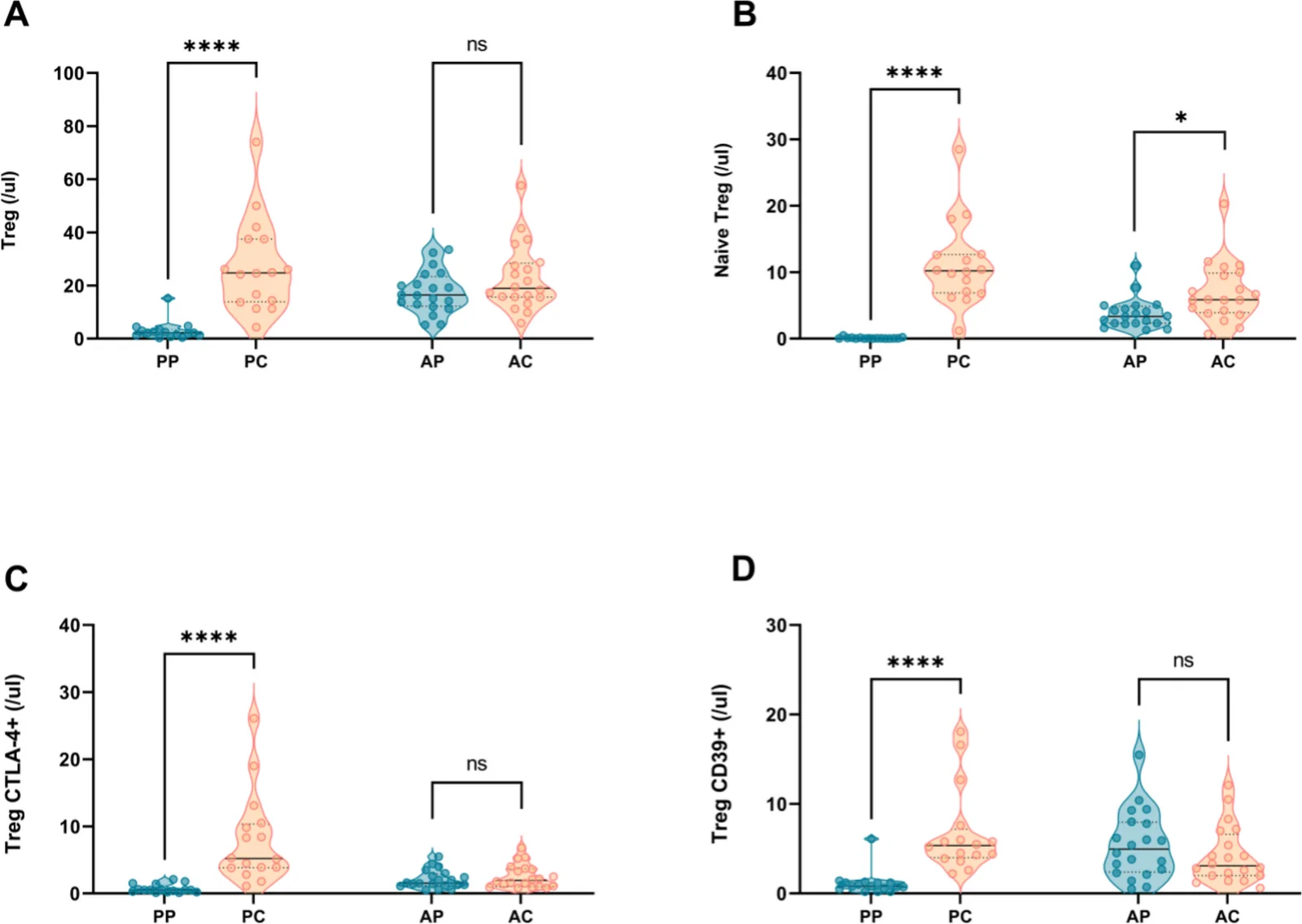
Regulatory T-cell subsets in thymectomized patients and healthy controls. Violin plots with individual data points showing (**A**) total regulatory T cells (Tregs), (**B**) naïve Tregs, (**C**) CTLA-4⁺ Tregs, and (D) CD39⁺ Tregs in pediatric thymectomized patients (PP), pediatric controls (PC), adult thymectomized patients (AP), and adult controls (AC). Values are expressed as cells/µL. Horizontal lines indicate summary measures within each group. Statistical comparisons were performed between thymectomized patients and their respective age-matched controls using FDR-adjusted p-values. *p < 0.05; ****p < 0.0001; ns, not significant

Individual-value plots of total Tregs, naïve Tregs, CTLA-4⁺ Tregs, and CD39⁺ Tregs are shown in Fig. 2, highlighting the marked reduction in regulatory T-cell subsets, particularly in pediatric thymectomized patients.

## Discussion

This study demonstrates long-term immunological alterations in the T-cell compartment of pediatric and adult patients with CHD who underwent early thymectomy. To our knowledge, this is the first Latin American cohort to assess both thymic output and detailed T-cell immunophenotyping in this population. Notably, the adult cohort included individuals in the third and fourth decades of life, representing one of the longest post-thymectomy follow-up cohorts with extensive T-cell subset analysis reported to date.

Our results are consistent with a previous systematic review of 23 studies evaluating immune outcomes in patients with CHD who underwent early thymectomy during surgical repair [14]. In both pediatric and adult patients, we observed a significant reduction in TREC levels, supporting long-lasting impairment of thymic output, independently demonstrated in the pediatric and adult cohorts. This was accompanied by lower circulating lymphocyte counts, largely driven by reductions in the naïve T-cell compartment. In contrast, previous studies have reported more heterogeneous findings regarding non-naïve and Treg compartments. In our cohort, most non-naïve subsets, including effector, memory, senescence-associated, and exhaustion-associated T cells, did not differ significantly from controls, a pattern that may be consistent with compensatory peripheral expansion. The marked reduction in Tregs observed in pediatric patients was not uniformly described in previous studies, highlighting the variability of this compartment after early thymectomy [14]. More recent high-dimensional analyses have further expanded the spectrum of immune alterations after early-life thymectomy in children with CHD, showing perturbations of the γδ T-cell compartment, including expansion of granzyme-producing γδ T cells and changes in γδ T-cell repertoire composition [15].

The reduction in TREC copy numbers observed in both pediatric and adult patients in the present study supports the concept that impaired thymic output after early thymectomy is not merely a transient phenomenon restricted to the first years after surgery. Most previous studies have focused on children, either prospectively after surgery or through cross-sectional analyses, and have consistently reported reduced TREC levels in patients who underwent thymectomy during cardiac surgery [7, 12, 13, 16–18]. Reduced TREC levels beyond childhood have also been reported in exclusively adult populations [19, 20], as well as in cohorts including both children and adults [11, 21, 22]. In contrast, a study from the Netherlands [23] found reduced TREC levels compared with controls only during the first 5 years after early thymectomy, with normalization thereafter, suggesting a possible role for thymic regeneration. However, this pattern has not been consistently replicated in other populations, including the present cohort.

Consistent with the biological role of the thymus in T-cell generation, the reduction in total lymphocyte counts observed in pediatric and adult patients appeared to be mainly driven by diminished numbers of naïve T-cell subsets, including CD4⁺, CD8⁺, and naïve Treg subsets. Importantly, CD31-expressing naïve CD4⁺ recent thymic emigrants (RTE) are also considered relevant markers of recent thymic output and have been shown to correlate with TREC levels [24, 25]. Therefore, the significantly lower numbers of CD4⁺ RTEs observed in our cohort further corroborate the finding of impaired thymic function.

When analyzing the broader memory T-cell compartment (CD3⁺CD45RO⁺), no significant difference was observed between adult patients and controls, whereas pediatric patients showed a statistically significant reduction. However, the magnitude of this reduction was smaller than that observed for naïve T cells (CD3⁺CD45RA⁺). When the analysis was expanded to non-naïve CD4⁺ and CD8⁺ T-cell subsets, heterogeneous findings were observed, with most subsets not differing significantly from controls in either age group. This heterogeneity is consistent with previous studies, which have reported either preserved [7, 13, 23] or reduced [8, 21, 26] memory T-cell compartments in patients who underwent early thymectomy compared with controls. Notably, most previous studies did not evaluate such a broad range of non-naïve T-cell subsets. Since total lymphocyte and T-cell counts were reduced in both age groups, mainly because of decreased naïve T-cell subsets, while most non-naïve subsets were preserved or less markedly reduced, the relative contribution of memory T cells to the remaining T-cell compartment was likely higher in thymectomized patients. These findings may reflect compensatory peripheral proliferation in response to reduced thymic naïve T-cell output, although this mechanism was not directly assessed in the present study using functional or proliferation markers such as Ki-67 [27]. In line with this interpretation, a recent functional profiling study in children with CHD after early-life thymectomy reported persistent αβ T-cell alterations, including reduced naïve T cells and recent thymic emigrants, together with accumulation of memory/effector and senescent-like T cells and evidence of ongoing T-cell activation [28].

The evaluation of regulatory T cells has yielded heterogeneous results in previous studies, but these findings appear to vary according to the age group evaluated. Studies reporting reduced Treg counts in patients with CHD who underwent early thymectomy were performed in pediatric populations [9, 13, 29, 30], whereas studies showing no significant differences between patients and controls included adults [10, 19]. In our adult patients, we observed a selective reduction in naïve Tregs, while total Tregs and Treg subsets expressing suppression-associated markers (CTLA-4⁺ and CD39⁺) did not differ significantly from controls, possibly reflecting peripheral differentiation and/or homeostatic maintenance of the Treg compartment, although this was not directly assessed. Indeed, although most Tregs are generated in the thymus, a smaller proportion may arise from conventional CD4⁺ T cells in secondary lymphoid tissues [31]. Conversely, pediatric patients in our cohort showed not only a marked reduction in naïve Tregs, but also significantly lower counts of total Tregs and CTLA-4⁺ and CD39⁺ Treg subsets. This finding is consistent with previous pediatric studies and with the observation by van den Broek et al. that Treg counts were reduced in patients younger than 5 years after neonatal thymectomy, whereas older patients (9–29 years) showed no significant differences compared with controls [26]. Taken together, these data support the hypothesis that Treg alterations after early thymectomy may be more pronounced earlier in life and partially compensated over time.

Considering the cross-sectional design of the study and the limited number of individuals in each group, clinical outcomes such as infections, autoimmunity, and malignancy were not systematically analyzed, as such evaluations would be highly susceptible to recall, selection, and survival biases. Patients with previous diagnoses of inborn errors of immunity or clinical features suggestive of 22q11.2 deletion/DiGeorge syndrome, trisomy 21, or other syndromic conditions were excluded; however, genetic testing was not available for the participants, and therefore underlying genetic conditions cannot be completely ruled out. In addition, because adult and pediatric patients have different age-related reference ranges for lymphocyte subsets and underwent cardiac surgery in different historical periods, direct statistical comparisons of immunological outcomes between these age groups were not performed. Because genetic ancestry was not assessed and self-reported race/color or ethnicity was not systematically collected, we cannot determine the contribution of European, African, Indigenous/Native American, or other ancestral backgrounds to the observed immune phenotype. Given the highly admixed nature of the Brazilian population, the term Brazilian/Latin American refers to the geographic origin of recruitment rather than genetically defined ancestry. Finally, additional limitations include the lack of functional immune assays, such as proliferative responses, Ki-67 expression, TCR repertoire analysis, or telomere length assessment.

Since more than 97% of children with CHD are currently expected to reach adulthood [32], and a substantial proportion of those with complex CHD requiring early surgical repair may undergo thymectomy to facilitate surgical exposure, identifying potential long-term health risks in this growing population has become increasingly important. Historically, an overt clinical immunodeficiency phenotype has not been clearly associated with early thymectomy [33]. More recently, limited population-based data have suggested a possible increase in the risk of selected autoimmune diseases, infections, and respiratory conditions after early thymectomy [34, 35]. Further prospective longitudinal studies with larger cohorts, as well as national registry-based analyses, are needed to clarify the actual clinical risks associated with early thymectomy, particularly later in life.

## Conclusion

Early thymectomy during surgical repair of complex congenital heart disease was associated with markedly reduced thymic output and sustained depletion of naïve T-cell subsets in both children and adults. The broader reduction in total Tregs and Treg subsets expressing suppression-associated markers in children, contrasted with the selective reduction in naïve Tregs in adults, suggests age-dependent remodeling or peripheral maintenance of the regulatory T-cell compartment after thymectomy. Although most non-naïve T-cell subsets were relatively maintained, the clinical significance of these long-lasting immunological alterations remains uncertain. Prospective longitudinal and population-based studies are needed to determine whether these changes translate into increased risks of infection, autoimmunity, malignancy, or other immune-mediated outcomes.

## Supplementary Information

Below is the link to the electronic supplementary material.

Supplementary file1 (DOCX 16 KB)

Supplementary file2 (DOCX 42 KB)

## Data Availability

The de-identified individual-level data underlying this article, including immunophenotyping and T-cell receptor excision circle measurements, are available in Mendeley Data at https://doi.org/10.17632/zymhny9wg3.1.
