# Supplementary material for: Long-term immunological impairment after early thymectomy in congenital heart disease
Source: Immunol Res. 2026 Oct 7;74(1):122. doi: 10.1007/s12026-026-09858-z (PMC13645957; doi:10.1007/s12026-026-09858-z)
Supplement: Supplementary file 1 [file 12026_2026_9858_MOESM1_ESM.docx]

| **Monoclonal antibody** | **Fluorochrome** | **Clone** | **Volume (µL)** |
| --- | --- | --- | --- |
| **T Lymphocytes** | | | |
| CD45RA | PE-Cy7 | L48 | 4.0 |
| CD27 | V500 | M-T271 | 3.0 |
| CD3 | BUV395 | SK7 | 2.0 |
| CD62L | BV786 | SK11 | 2.0 |
| HLA-DR | PE-Cy5 | L243 | 4.0 |
| CCR7 | PE | 3D12 | 2.0 |
| CD31 | APC-Cy7 | WM59 | 2.0 |
| CD45RO | BV605 | UCHL1 | 2.0 |
| CD8 | FITC | SK1 | 3.0 |
| CD4 | PE-CF594 | RPA-T4 | 1.5 |
|  |  |  |  |
| **Senescence and Exhaustion T** **Lymphocytes** | | | |
| CD28 | PE-Cy5 | CD28.2 | 4.0 |
| CD45RA | PE-Cy7 | L48 | 4.0 |
| CD27 | V500 | M-T271 | 2.0 |
| CD45RO | BV605 | UCHL1 | 2.0 |
| CD3 | BUV395 | SK7 | 2.0 |
| CD57 | BB515 | NK-1 | 2.0 |
| PD-1 (CD279) | APC | MIH4 | 2.0 |
| CD8 | FITC | SK1 | 3.0 |
| CD4 | PE-CF594 | RPA-T4 | 1.5 |
|  |  |  |  |
| **Treg cells** | | | |
| CD152 (CTLA-4) | PE-Cy5 | BNI3 | 4.0 |
| CD127 | BV421 | HIL-7R-M21 | 4.0 |
| CD45RA | PE-Cy7 | L48 | 4.0 |
| CD25 | APC-Cy7 | M-A251 | 4.0 |
| CD39 | APC | TU66 | 3.0 |
| CD3 | BUV395 | SK7 | 2.0 |
| CD4 | PE-CF594 | RPA-T4 | 1.5 |
| FOXP3 | PE | PCH101 | 1.5 |

**Supplementary Table S1.** Monoclonal Antibodies Used for the Immunophenotypic Characterization of T Lymphocyte Subsets, Senescence and Exhaustion Markers, and Regulatory T Cells
