# Supplementary material for: Long-term immunological impairment after early thymectomy in congenital heart disease
Source: Immunol Res. 2026 Oct 7;74(1):122. doi: 10.1007/s12026-026-09858-z (PMC13645957; doi:10.1007/s12026-026-09858-z)
Supplement: Supplementary file 2 [file 12026_2026_9858_MOESM2_ESM.docx]

**Supplementary Table S2. Detailed lymphocyte immunophenotyping with raw and FDR-adjusted p-values.**

**Supplementary Table S2A. Total lymphocytes, T cells, and major T-cell subsets**

| Lymphocyte subset  (phenotypic markers) | AP  (n=20) | AC  (n=20) | Raw p | FDR-adjusted p | PP  (n=16) | PC  (n=16) | Raw p | FDR-adjusted p |
| --- | --- | --- | --- | --- | --- | --- | --- | --- |
|  |  |  |  |  |  |  |  |  |
| Total lymphocytes | 1790  ± 453 | 2275  ± 504 | 0.003 | **<0.01** | 1806  ± 643 | 2682  ± 627 | 0.0005 | **0.001** |
|  |  |  |  |  |  |  |  |  |
| Total T cells  (CD3⁺) | 1019  ± 333 | 1523  ± 409 | 0.0001 | **<0.001** | 870  (660–1055) | 1558  (1350–1709) | 0.000003 | **<0.0001** |
| Naïve T cells  (CD3⁺CD45RA⁺) | 350  ± 152 | 670  ± 204 | 0.000002 | **<0.0001** | 340  ± 202 | 955  ± 315 | 0.00000004 | **<0.0001** |
| RTE T cells  (CD3⁺CD45RA⁺CD27⁺  CCR7⁺CD62L⁺CD31⁺) | 72  ± 73 | 251  ± 105 | 0.0000003 | **<0.0001** | 77  (23–142) | 339  (262–404) | 0.0002 | **<0.001** |
| Mature naïve T cells  (CD3⁺CD45RA⁺CD27⁺  CCR7⁺CD62L⁺CD31⁻) | 33  (10–82) | 105  (59–139) | 0.002 | **<0.01** | 60  ± 51 | 165  ± 80 | 0.0001 | **<0.001** |
| Memory T cells  (CD3⁺CD45RO⁺) | 522  ± 178 | 589  ± 247 | *0.33* | *0.42* | 388  (286–526) | 536  (470–761) | 0.004 | **<0.01** |

All values are expressed as cells/μL. Data are presented as mean ± SD or median (IQR), according to distribution. Raw p-values and Benjamini–Hochberg false discovery rate (FDR)–adjusted p-values are shown. Statistical significance was defined as FDR-adjusted p < 0.05. AP, adult patients; AC, adult controls; PP, pediatric patients; PC, pediatric controls; RTE, recent thymic emigrant.

**Supplementary Table S2B. CD4⁺ and CD8⁺ T cells and naïve subsets**

| Lymphocyte subset  (phenotypic markers) | AP  (n=20) | AC  (n=20) | Raw p | FDR-adjusted p | PP  (n=16) | PC  (n=16) | Raw p | FDR-adjusted p |
| --- | --- | --- | --- | --- | --- | --- | --- | --- |
|  |  |  |  |  |  |  |  |  |
| Total CD4⁺ T cells  (CD3⁺CD4⁺) | 577  ± 181 | 804  ± 269 | 0.003 | **<0.01** | 481  (364–524) | 809  (693–909) | 0.00002 | **<0.0001** |
| Naïve CD4⁺ T cells  (CD3⁺CD4⁺CD45RA⁺) | 144  (108–176) | 293  (229–347) | 0.0005 | **<0.01** | 80  (53–135) | 436  (367–518) | 0.000002 | **<0.0001** |
| CD4⁺ naïve RTE T cells  (CD3⁺CD4⁺CD45RA⁺CD27⁺  CCR7⁺CD62L⁺CD31⁺) | 30  (8–56) | 100  (76–161) | 0.00005 | **<0.001** | 40  ± 40 | 169  ± 94 | 0.00002 | **<0.0001** |
| CD4⁺ mature naïve T cells  (CD3⁺CD4⁺CD45RA⁺CD27⁺  CCR7⁺CD62L⁺CD31⁻) | 37  (9–59) | 84  (37–107) | 0.003 | **<0.01** | 21  (11–43) | 95  (80–103) | 0.0004 | **<0.001** |
|  |  |  |  |  |  |  |  |  |
| Total CD8⁺ T cells  (CD3⁺CD8⁺) | 244  (185–330) | 453  (403–530) | 0.00005 | **<0.001** | 288  (170–351) | 460  (357–512) | 0.0003 | **<0.001** |
| Naïve CD8⁺ T cells  (CD3⁺CD8⁺CD45RA⁺) | 103  (72–137) | 209  (161–294) | 0.00004 | **<0.001** | 114  (38–162) | 236  (180–300) | 0.00001 | **<0.0001** |

All values are expressed as cells/μL. Data are presented as mean ± SD or median (IQR), according to distribution. Raw p-values and Benjamini–Hochberg false discovery rate (FDR)–adjusted p-values are shown. Statistical significance was defined as FDR-adjusted p < 0.05. AP, adult patients; AC, adult controls; PP, pediatric patients; PC, pediatric controls; RTE, recent thymic emigrant.

**Supplementary Table S2C. Non-naïve CD4⁺ and CD8⁺ T-cell subsets**

| Lymphocyte subset  (phenotypic markers) | AP  (n=20) | AC  (n=20) | Raw p | FDR-adjusted p | PP  (n=16) | PC  (n=16) | Raw p | FDR-adjusted p |
| --- | --- | --- | --- | --- | --- | --- | --- | --- |
|  |  |  |  |  |  |  |  |  |
| Activated CD4⁺ T cells  (CD3⁺CD4⁺HLA-DR⁺) | 45  (33–66) | 51  (36–75) | *0.66* | *0.70* | 25  (11–74) | 34  (21–42) | *0.7* | *0.74* |
| CD4⁺ memory T cells  (CD3⁺CD4⁺CD45RO⁺) | 318  ± 111 | 372  ± 138 | *0.18* | *0.28* | 292  ± 90 | 369  ± 130 | *0.06* | *0.08* |
| CD4⁺ TCM  (CD3⁺CD4⁺CD45RO⁺  CD27⁺CCR7⁺CD62L⁺) | 74  ± 38 | 103  ± 62 | *0.08* | *0.15* | 132  ± 55 | 123  ± 62 | *0.64* | *0.71* |
| CD4⁺ TTM  (CD3⁺CD4⁺CD45RO⁺  CD27⁺CCR7⁻CD62L⁻) | 21  (15–30) | 32  (19–48) | *0.19* | *0.28* | 12  (10–18) | 22  (13–26) | 0.01 | **0.02** |
| CD4⁺ TEM  (CD3⁺CD4⁺CD45RO⁺  CD27⁻CCR7⁻CD62L⁻) | 47  (30–59) | 52  (37–67) | *0.48* | *0.54* | 26  ± 15 | 48  ± 29 | 0.01 | **0.02** |
| CD4⁺ TEMRA cells  (CD3⁺CD4⁺CD45RA⁺CCR7⁻) | 41  (21–55) | 56  (31–94) | *0.12* | *0.20* | 19  (9–28) | 71  (46–114) | 0.00004 | **0.0001** |
| Senescence CD4⁺ T cells  (CD3⁺CD4⁺CD45RO⁻CD27⁻CD28⁻CD57⁺) | 4  (2.8–12.5) | 4.1  (1.7–7) | *0.48* | *0.54* | 2.7  (0.7–8.7) | 3.5  (1.1–8.2) | *0.64* | *0.71* |
| Exhaustion-associated CD4⁺ memory T cells  (CD3⁺CD4⁺CD45RO⁺  CD27⁻PD-1⁺) | 95  (64–139) | 96  (55–159) | *0.95* | *0.95* | 45  (28–50) | 37  (15–44) | *0.30* | *0.36* |
|  |  |  |  |  |  |  |  |  |
| Activated CD8⁺ T cells  (CD3⁺CD8⁺HLA-DR⁺) | 27  (15–58) | 37  (27–49) | *0.19* | *0.28* | 16  (6–28) | 20  (14–33) | *0.21* | *0.25* |
| CD8⁺ memory T cells  (CD3⁺CD8⁺CD45RO⁺) | 107  (80–157) | 179  (111–210) | 0.005 | **0.01** | 117  (89–143) | 164  (125–199) | 0.02 | **0.03** |
| CD8⁺ TCM  (CD3⁺CD8⁺CD45RO⁺CD27⁺  CCR7⁺CD62L⁺) | 12  (9–26) | 17  (9–34) | *0.46* | *0.54* | 18  (12–32) | 31  (24–40) | *0.06* | *0.08* |
| CD8⁺ TTM  (CD3⁺CD8⁺CD45RO⁺CD27⁺  CCR7⁻CD62L⁻) | 15  (8–22) | 21  (17–37) | 0.007 | **0.02** | 12  (10–20) | 14  (7–20) | *0.98* | *0.98* |
| CD8⁺ TEM  (CD3⁺CD8⁺CD45RO⁺CD27⁻  CCR7⁻CD62L⁻) | 14  (3–19) | 22  (11–35) | 0.04 | *0.09* | 7.7  (4.7–16.1) | 16  (8–26) | 0.03 | **0.04** |
| CD8⁺ TEMRA cells  (CD3⁺CD8⁺CD45RA⁺CCR7⁻) | 69  ± 44 | 116  ± 70 | 0.02 | **0.03** | 52  (14–89) | 101  (63–154) | 0.004 | **<0.01** |
| Senescence CD8⁺ T cells  (CD3⁺CD8⁺CD45RO⁻CD27⁻  CD28⁻CD57⁺) | 27  (13–77) | 30  (10–71) | *0.92* | *0.95* | 16  (5–28) | 29  (10–43) | *0.13* | *0.16* |
| Exhaustion-associated CD8⁺ memory T cells  (CD3⁺CD8⁺CD45RO⁺  CD27⁻PD-1⁺) | 43  (25–76) | 32  (19–65) | *0.33* | *0.42* | 14  (5–21) | 7.8  (5.6–18) | *0.84* | *0.86* |

All values are expressed as cells/μL. Data are presented as mean ± SD or median (IQR), according to distribution. Raw p-values and Benjamini–Hochberg false discovery rate (FDR)–adjusted p-values are shown. Statistical significance was defined as FDR-adjusted p < 0.05. AP, adult patients; AC, adult controls; PP, pediatric patients; PC, pediatric controls; TCM, central memory T cells; TTM, transitional memory T cells; TEM, effector memory T cells; TEMRA, terminally differentiated effector memory T cells re-expressing CD45RA.

**Supplementary Table S2D. Regulatory T-cell subsets**

| Lymphocyte subset  (phenotypic markers) | AP  (n=20) | AC  (n=20) | Raw p | FDR-adjusted p | PP  (n=16) | PC  (n=16) | Raw p | FDR-adjusted p |
| --- | --- | --- | --- | --- | --- | --- | --- | --- |
|  |  |  |  |  |  |  |  |  |
| Total Tregs  (CD3⁺CD4⁺CD127⁻  CD25⁺FoxP3⁺) | 18  ± 8 | 23  ± 12 | *0.12* | *0.20* | 2.2  (1.3–3.2) | 25  (14–37) | 0.0000002 | **<0.0001** |
| Naïve Tregs  (CD3⁺CD4⁺CD127⁻  CD25⁺FoxP3⁺CD45RA⁺) | 3.3  (2.3–4.7) | 5.9  (4–9.8) | 0.004 | **0.01** | 0.04  (0.02–0.12) | 10.2  (6.8–12.6) | *0.000000003* | **<0.0001** |
| CTLA-4⁺ Tregs  (CD3⁺CD4⁺CD127⁻  CD25⁺FoxP3⁺CTLA-4⁺) | 1.6  (1.2–2.8) | 1.9  (1–3.7) | *0.56* | *0.62* | 0.5  (0.2–1) | 5.2  (3.8–9.7) | *0.00000006* | **<0.0001** |
| CD39⁺ Tregs  (CD3⁺CD4⁺CD127⁻  CD25⁺FoxP3⁺CD39⁺) | 4.9  (2.5–7.9) | 3.1  (2–6.2) | *0.30* | *0.42* | 0.8  (0.5–1.2) | 5.3  (3.9–6) | *0.0000009* | **<0.0001** |

All values are expressed as cells/μL. Data are presented as mean ± SD or median (IQR), according to distribution. Raw p-values and Benjamini–Hochberg false discovery rate (FDR)–adjusted p-values are shown. Statistical significance was defined as FDR-adjusted p < 0.05. AP, adult patients; AC, adult controls; PP, pediatric patients; PC, pediatric controls; Tregs, regulatory T cells.
